# The New Era of Immunotherapy in Gastric Cancer

**DOI:** 10.3390/cancers14041054

**Published:** 2022-02-18

**Authors:** Shogo Takei, Akihito Kawazoe, Kohei Shitara

**Affiliations:** Department of Gastrointestinal Medical Oncology, National Cancer Center Hospital East, Chiba 277-8577, Japan; shtakei@east.ncc.go.jp (S.T.); akawazoe@east.ncc.go.jp (A.K.)

**Keywords:** gastric cancer, immunotherapy, immune checkpoint inhibitors, chemotherapy, programmed cell death-1, programmed cell death ligand-1

## Abstract

**Simple Summary:**

Advanced gastric cancer remains a malignancy with a poor prognosis, with a median survival of about 12–15 months. In recent years, immune checkpoint inhibitors have emerged as a new standard of care for several malignancies, including advanced gastric cancer, and have demonstrated good clinical benefit in some populations. In this review paper, we describe the current status of immunotherapy in gastric cancer, with a focus on molecular and immunological profiles, biomarkers, major clinical trials, and novel immunotherapies.

**Abstract:**

Immune checkpoint inhibitors (ICIs) such as anti-programmed cell death-1 (PD-1) or programmed cell death ligand-1 (PD-L1) monoclonal antibodies have prolonged survival in various types of malignancies, including advanced gastric cancer (AGC). Nivolumab, a monoclonal anti-PD-1 antibody, showed an improvement in overall survival at a later-line therapy in unselected AGC patients in the ATTRACTION-2 study or in combination with chemotherapy as first-line therapy in the global CheckMate-649 study. Another monoclonal anti-PD-1 antibody, pembrolizumab, showed single agent activity in tumors with high microsatellite instability or high tumor mutational burden. Furthermore, a recent KEYNOTE-811 study demonstrated significant improvement in response rate with pembrolizumab combined with trastuzumab and chemotherapy for HER2-positive AGC. Based on these results, ICIs are now incorporated into standard treatment for AGC patients. As a result of pivotal clinical trials, three anti-PD-1 antibodies were approved for AGC: nivolumab combined with chemotherapy as first-line treatment or nivolumab monotherapy as third- or later-line treatment in Asian countries; pembrolizumab for previously treated microsatellite instability-high (MSI-H) or tumor mutational burden-high AGC, or pembrolizumab combined with trastuzumab and chemotherapy for HER2-positive AGC in the United States; and dostarlimab for previously treated MSI-H AGC in the United States. However, a substantial number of patients have showed resistance to ICIs, highlighting the importance of the better selection of patients or further combined immunotherapy. This review focused on molecular and immunological profiles, pivotal clinical trials of ICIs with related biomarkers, and investigational immunotherapy for AGC.

## 1. Introduction

**Figure 1:**
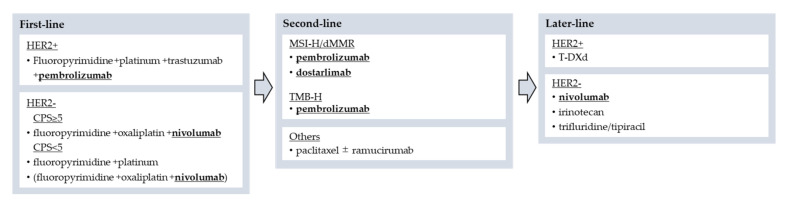
This figure shows current position of immunotherapy for advanced gastric cancer. Abbreviations: CPS: PD-L1 combined positive score; T-DXd: trastuzumab deruxtecan.

Gastric cancer is the fourth leading cause of cancer death in the world and the fifth most common malignant tumor [1]. Combination regimens, including a fluoropyrimidine and a platinum agent (plus trastuzumab as an anti HER2 monoclonal antibody for HER2-positive cases) at first-line and paclitaxel with or without ramucirumab at second-line, are standard treatment for advanced unresectable or recurrent gastric cancer (AGC). However, the median survival of AGC is still approximately 12–15 months and introduction of newer treatment is required [2,3,4,5]. Recently, immune checkpoint inhibitors (ICIs) have emerged as new standard treatment in several malignancies, including AGC with favorable clinical benefit in some populations [6,7,8,9,10]. In AGC, pembrolizumab, a humanized IgG4 monoclonal antibody against programmed cell death-1 (PD-1), can be used as second-line or subsequent treatment for patients with microsatellite instability-high (MSI-H) or tumor mutational burden-high (TMB-H) [11,12]. Most recently, based on the interim results of KEYNOTE-811, pembrolizumab received accelerated approval by the Food and Drug Administration (FDA) in combination with trastuzumab, first-line chemotherapy for patients with HER2 positive AGC [13]. Another anti-PD-1 antibody, nivolumab, showed a survival benefit in third-line or subsequent treatment in an Asian patient population irrespective of PD-L1 expression (ATTRACTION-2) or in first-line treatment combined with standard cytotoxic agents (CheckMate-649) [14,15]. On the other hand, dostarlimab, an anti-PD-1 antibody, demonstrated a favorable ORR in the GARNET trial in MMR-D patients with non-endometrial solid tumors [16], and was granted accelerated approval by the FDA. Furthermore, clinical trials of several investigational immunotherapies are ongoing in AGC, including anti-PD-1 antibody plus anti-CTLA4 antibody, ICIs plus other targeted agents, and chimeric antigen receptor T (CAR-T) cell therapies. In this review, we will discuss current status of immunotherapy for gastric cancer (Figure 1), including molecular and immunological profiles, pivotal clinical trials of ICIs with related biomarkers, and investigational immunotherapy.

## 2. Molecular and Immunological Profiles in Gastric Cancer

In 2014, the Cancer Genome Atlas (TCGA) study proposed four molecular subtypes of gastric cancer: Epstein–Barr virus (EBV), MSI, chromosomal instability (CIN), and genomically stable (GS), based on analysis of somatic copy numbers, whole-exome sequencing (WES), DNA methylation profiling, messenger RNA sequencing, microRNA sequencing, and reverse-phase protein array [17]. EBV-positive tumors have the poorly differentiated adenocarcinoma, with a high content of immune cells and high expression of PD-L1 and PD-L2 [18,19,20,21,22]. In the TCGA study, EBV-positive tumors exhibit recurrent *PIK3CA* and *ARIDIA* mutations, extreme DNA hypermethylation and high amplifications of *JAK2*, PD-L1, and PD-L2. MSI-H tumors exhibit elevated mutation rates (including frameshifts or missense mutations) and hypermethylation (including hypermethylation at the MLH1 promoter), resulting in the enhanced expression of neoantigens [17]. Consequently, MSI-H tumors display high infiltration with CD8+ T cells, presumably due to the recognition of a high number of neoantigens and its corresponding expression of immune checkpoints, such as PD-L1 in the tumor microenvironment [23]. GS tumors are typically enriched for the diffuse histology and mutations of *CDH1* and *RHOA* or *CLDN18–ARHGAP* fusion [24,25,26,27]. CIN tumors are frequently observed at the gastroesophageal junction/cardia with recurrent *TP53* mutation and relatively high amplifications of receptor tyrosine kinase (RTKs) genes [17]. Transcriptomic analysis in the TCGA study demonstrated the significant upregulation of immune cell signaling in the EBV-positive or MSI-H subtypes compared with the GS or CIN subtypes [28].

In stage IV AGC, EBV-positive and mismatch repair (MMR)-deficient (MMR-D) tumors are identified in 6.2% and 6.2% cases, respectively [22]. As mentioned above, EBV-positive or MSI-H tumors have distinct immunological profiles, which might lead to a favorable response to ICIs [29,30]. Moreover, MSI-H/MMR-D AGC patients have been reported to be associated with shorter progression-free survival (PFS) on first-line cytotoxic chemotherapy, but achieved durable response from subsequent anti-PD-1 therapy [30]. Recently, the majority of CIN tumors have been reported to exhibit T cells’ exclusion and infiltrating CD68+ macrophages [31]. GS tumors showed enrichment of CD4+ T cells, tumor-associated macrophages, and B cells, and half of cases displayed tertiary lymphoid structures [31]. Thus, targeting immune-suppressive macrophages or other upregulated pathways might enhance ICIs in the CIN or GS subtypes.

Analysis of more than 1000 gastric cancer samples demonstrated that in comparison with Asian tumors, non-Asian gastric cancers had higher expression of T cell markers (CD3, CD45R0, and CD8), including CTLA-4 signaling and lower expression of the immunosuppressive T regulatory cell marker FOXP3 [32]. These differences in immunological profiles warrant further investigation, together with comparison of response to ICIs between the Asian and non-Asian population.

PD-L1 combined positive score (CPS), which has been defined as number of PD-L1-positive cells, including tumor cells, macrophages, and lymphocytes, divided by the total number of tumor cells and multiplied by 100, is currently used for selection of ICIs in several malignancies such as AGC [15,33,34,35,36,37,38]. Impact of CPS on clinical outcomes with ICIs in AGC will be described in the next session.

## 3. Clinical Trials of Immunotherapy for Gastric Cancer with Related Biomarkers (PD-L1, MSI, and TMB)

Table 1 summarizes 18 major clinical trials for immunotherapy for AGC: 8 for anti-PD-1/PD-L1 monotherapy, 4 for anti-PD-1/PD-L1 antibody plus chemotherapy, 2 for anti-PD-1 antibody plus HER2-targeted therapy, 2 for anti-PD-1 antibody plus anti-CTLA4 antibody, 3 for anti-PD-1 antibody plus multikinase inhibitors, and 1 trial for chimeric antigen receptor T (CAR-T) cell therapies.

### 3.1. Anti-PD-1/PD-L1 Monotherapy

In the phase III ATTRACTION-2 trial, nivolumab as an anti-PD-1 monoclonal antibody improved overall survival (OS) compared with the placebo in patients with AGC after two or more previous lines of chemotherapy (median OS 5.26 months vs. 4.14 months; hazard ratio (HR) = 0.63; *p* < 0.0001) [14]. PFS (median 1.61 months vs. 1.45 months; HR ≥ 0.60; *p* < 0.0001) and objective response rate (ORR) (11.2% vs. 0%; *p* < 0.0001) were also improved with nivolumab. These results led to the approval of nivolumab for AGC in Asian countries. An exploratory analysis of ATTRACTION-2 suggested no relationship between survival benefit and PD-L1 expression on tumor cells, although tumor samples were available from less than 40% of patients. Any-grade treatment-related adverse events (TRAEs) had occurred in 43% of patients treated with nivolumab, including 10% grade 3 or 4 events. All-grade TRAEs reported in 5% or more of patients with nivolumab were pruritus (9%), diarrhea (7%), rash (6%), and fatigue (5%). Common grade 3 or 4 TRAEs with nivolumab included decreased appetite, diarrhea, fatigue, and increased aspartate transaminase. Another anti-PD-1 monoclonal antibody, pembrolizumab, showed ORR of 11.6% at third-line or later-line in a phase II KEYNOTE-059 trial [34]. ORR was 15.5% for patients with PD-L1-positive tumors (CPS ≥ 1) as assessed by 22C3 IHC assay, while ORR was 6.4% for those with CPS < 1. Meanwhile, the global phase III trial (JAVELIN 300) of avelumab (anti-PD-L1 antibody) failed to show an OS improvement compared with investigators’ choice of third-line chemotherapy, which included paclitaxel or irinotecan in patients with AGC [39].

In the second-line setting (KEYNOTE-061), pembrolizumab did not significantly improve PFS and OS compared with paclitaxel in patients with PD-L1 CPS ≥ 1 [33]. In the first-line setting (KEYNOTE-062), pembrolizumab was non-inferior to chemotherapy for OS in patients with CPS ≥ 1 [36]. The crossing of survival curves in both the KEYNOTE-061 and KEYNOTE-062 suggested that some patients treated with pembrolizumab had early disease progression with poor prognosis. Exploratory analyses of these trials found the trend toward better clinical outcomes in patients with MSI-H or high PD-L1 expression (CPS ≥ 10) to suggest that these biomarkers may be useful for better selection of patients who might derive greater benefit from PD-1 blockade immunotherapy [38,40]. Although maintenance avelumab monotherapy was compared with continued chemotherapy or best supportive care after induction of first-line treatment in the phase III trial of avelumab (JAVELIN 100), it failed to show superior OS, either in all randomized patients or in a PD-L1-positive (≥1% of tumor cells) patient cohort [41]. In an exploratory subset analysis, a CPS ≥ 1 population by 22C3 assay showed a trend for longer OS with avelumab.

The FDA also granted accelerated approval to pembrolizumab for patients with previously treated MSI-H/MMR-D tumors, including AGC based on the durable response in several trials [11,42,43]. The phase II trial of pembrolizumab (KEYNOTE-158) showed ORR of 37.2% for 94 patients with MSI-H/MMR-D non-colorectal cancers [11]. In this trial, 11 of 24 patients with AGC achieved an objective response (ORR, 45.8%), with median PFS of 11.0 months and median OS was not reached. Moreover, a subgroup analysis of KEYNOTE-059, KEYNOTE-061, and KEYNOTE-062 showed consistent efficacies with ORR of 57%, 47%, and 57% for patients with MSI-H/MMR-D AGC, respectively [40]. In addition, post-hoc analysis of KEYNOTE-061 and KEYNOTE-062 suggested remarkable survival benefit for pembrolizumab in MSI-H patients. Most recently, the GARNET study (Cohort F) of dostarlimab (anti-PD-1 antibody) showed ORR of 38.7% in MMR-D patients with non-endometrial solid tumors (ORR of 37.5% for AGC) [16], leading to FDA accelerated approval to dostarlimab for advanced dMMR solid tumors with VENTANA MMR RxDx Panel. Currently, MSI-H/MMR-D is one of the most consistent predictive biomarkers for ICIs. However, approximately half of MSI-H/MMR-D patients did not respond to ICIs, highlighting the importance of identifying predictive biomarkers associated with unresponsiveness to these agents. Recently, our study revealed that TMB-low and *PTEN* mutations are predictors of a negative response to PD-1 blockade in 45 MSI-H/dMMR gastrointestinal tumors, including 18 AGC [44]. Interestingly, three of the four patients with TMB-low tumors had AGC, which might be due to geographic heterogeneity of MLH1 expression as previously reported [29]. Kwon and colleagues also reported that in the phase II trial of pembrolizumab in MSI-H AGC, non-responders had frequent mutations and upregulations in the Wnt/β-catenin pathway and an abundance of cancer-associated fibroblasts [45]. These findings might lead to further development of combination ICIs therapies for MSI-H/MMR-D AGC.

It is well known that TMB-high was associated with favorable clinical outcomes in patients receiving ICIs across multiple cancer types [46,47]. Indeed, in the phase II KEYNOTE-158 trial, TMB-high (defined as ≥10 mut/Mb using FoundationOne CDx™ assay) solid tumors with pembrolizumab were associated with higher ORR of 29% (28% after excluding MSI-H vs. non-TMB-high 6%) and higher 6-, 12-, and 24-month PFS rates compared with non-TMB-high [12]. Based on these results, pembrolizumab and FoundationOne CDx™ as a companion diagnostic assay have been approved by the FDA for patients with TMB-high solid tumors. Post-hoc biomarker analysis of KEYNOTE-061 and KEYNOTE-062 also demonstrated associations between TMB and favorable clinical efficacy with anti-PD-1 therapy, warranting further evaluation in other clinical trials, such as the CheckMate-649 trial [48,49].

### 3.2. Anti-PD-1/PD-L1 Antibody Plus Chemotherapy

Recently, outcomes of four pivotal phase III trials (KEYNOTE-062, ATTRACTION-4, CheckMate-649, and ORIENT-16), investigating the addition of anti-PD-1 antibodies to first-line chemotherapy for AGC, have been presented [15,36,50,51]. In the global KEYNOTE-062 trial, chemotherapy in combination with pembrolizumab fail to show benefits in OS and PFS in both CPS ≥ 1 and CPS ≥ 10 populations, although ORR was higher in pembrolizumab plus chemotherapy (49% vs. 37% in CPS ≥ 1) [36]. Recently, in the global CheckMate-649 trial of nivolumab plus chemotherapy (CapeOX or FOLFOX) compared with chemotherapy, met both primary endpoints of OS (median 14.4 months vs. 11.1 months; HR 0.70; *p* < 0.0001) and PFS (median 7.7 months vs. 6.0 months; HR 0.68; *p* <0.0001) in patients with PD-L1 CPS ≥ 5 using PD-L1 IHC 28–8 pharmDx assay (Dako) as well as the secondary endpoints of OS (median 14.0 months vs. 11.3 months; HR 0.77; *p* < 0.0001) in those with CPS ≥ 1 and OS (median 13.8 months vs. 11.6 months; HR 0.80; *p* ≥ 0.0002) in all randomized patients [15]. The unstratified HRs for OS with nivolumab plus chemotherapy versus chemotherapy were 0.92 in patients with CPS < 1 and 0.94 in those with CPS < 5, with significant interaction between OS and CPS observed at the cutoff of 5 (*p* ≥ 0.0107) but not at the cutoff of 1 (*p* ≥ 0.2041). Patients with nivolumab plus chemotherapy achieved higher ORR than in those with chemotherapy in the CPS ≥ 5 population (60% vs. 45%). Moreover, higher ORR was observed across PD-L1 CPS cutoffs, including CPS < 1 and <5. The magnitude of survival benefit was greater in patients with MSI-H tumors [15]. Meanwhile, in ATTRACTION-4 conducted in Asian countries without patient selection based on PD-L1 expression, adding nivolumab to chemotherapy (SOX or CapeOX) improved PFS (HR 0.68; *p* < 0.001) and ORR (58% vs. 48%) but not OS (HR 0.90; *p* ≥ 0.26) [50]. A higher proportion of patients in the control arm receiving subsequent therapy in ATTRACTION-4 (68% including 27% with ICIs) than in CheckMate-649 (41% including 8% with ICIs) might blur positive effects on OS in ATTRACTION-4. There was also a difference in the proportion of patients with gastroesophageal junction cancer between the two trials: about 8% in ATTRACTION-4 and about 18% in CheckMate-649, which also included about 12% of esophageal adenocarcinoma, although it may not be enough to explain the difference in OS results between two trials [15,52]. Most recently, in ORIENT-16 conducted in China, chemotherapy in combination with sintilimab (PD-1 inhibitor) was superior to chemotherapy for OS in both CPS ≥ 5 (median 18.4 vs. 12.9 months; HR 0.660; *p* ≥ 0.0023) and all randomized populations (median 15.2 vs. 12.3 months; HR 0.766; *p* ≥ 0.0090), with longer PFS and higher ORR [51]. In these four pivotal trials, grade 3 or 4 TRAEs increased by approximately 10% in anti-PD-1 antibodies plus chemotherapy compared with chemotherapy alone, but these are manageable. Based on the results of CheckMate-649, FDA approved the addition of nivolumab to standard chemotherapy (fluoropyrimidine and oxaliplatin) as the first-line treatment for AGC patients irrespective of PD-L1 CPS, but NCCN guidelines recommend it as a preferred regimen only for patients with PD-L1 CPS ≥ 5 [53]. In Asian countries (Japan, Korea, and China), nivolumab plus chemotherapy combination has been approved for AGC patients regardless of PD-L1 CPS. Meanwhile, the European Medicines Agency (EMA) approval is limited to patients with PD-L1 CPS ≥ 5. Japanese guidelines for the management of patients with metastatic gastric cancer have been stated as follows: (1) nivolumab plus chemotherapy (SOX, CapeOX or FOLFOX) is recommended as first-line therapy for AGC; (2) given that CPS was associated with survival outcomes in CheckMate-649, PD-L1 CPS evaluation should be conducted as much as possible, and (3) in cases where CPS < 5 or unknown, the decision to treat with nivolumab plus chemotherapy or chemotherapy alone should be taken with consideration to the patient’s overall fitness and access to subsequent therapies. Association between PD-L1 expression status and treatment efficacy of chemotherapy plus anti-PD-1 antibodies should also be investigated in ongoing phase III KEYNOTE-859 (NCT03675737) and BGBA317 305 (NCT03777657) trials.

In the second-line setting, a phase II/III trial of QL1604 (anti-PD-1 antibody) plus nab-paclitaxel versus paclitaxel is being tested (NCT04435652).

In mouse models of triple-negative breast cancer, neoadjuvant treatment with anti-PD-1 plus anti-CD137 monoclonal antibody has been shown to increase the number of cancer antigen-specific CD8+ T cells and improve survival compared with primary tumor resection followed by adjuvant treatment [54]. In fact, clinical trials have demonstrated that PD-1 inhibitors with or without conventional chemotherapy as a neoadjuvant setting have promising antitumor activity in several malignant tumors [55,56,57,58]. In gastric cancer, two phase III KEYNOTE-585 (NCT03221426) and MATTERHORN (NCT04592913) trials to evaluate adding anti-PD-1/PD-L1 to chemotherapy in a perioperative setting are ongoing. Additionally, in an adjuvant setting, a phase III trial, ATTRACTION-5 (NCT03006705), is underway to investigate standard adjuvant chemotherapy with S-1 or capecitabine plus oxaliplatin in combination of nivolumab for patients with pathological stage III gastric cancer (including esophagogastric junction cancer) after D2 or more extensive lymph node dissection.

### 3.3. Anti-PD-1 Antibody Plus HER2-Targeted Therapy

Several preclinical studies have indicated that trastuzumab increases HER2 internalization and cross-presentation by dendritic cells, upregulates PD-1 and PD-L1, induces expression of tumor-infiltrating lymphocytes, and modulates expression of major histocompatibility complex class II, resulting in the enhancement of HER2-specific T-cell responses [59,60,61]. In a HER2-positive immunocompetent mouse model, anti-PD-1 antibody could significantly improve antitumor activity of trastuzumab with the augment of antibody-dependent cellular cytotoxicity (ADCC) [62]. Phase II trials, evaluating the efficacy of first-line chemotherapy plus trastuzumab combined with pembrolizumab, have shown promising results with ORR of 76.7–91% and median PFS of 8.6–13.0 months [63,64]. The subsequent phase III KEYNOTE-811 trial of pembrolizumab plus trastuzumab and chemotherapy demonstrated a statistically significant 22.7% improvement in ORR in the pembrolizumab group compared with the placebo group (77.4% vs. 51.9%, *p* ≥ 0.00006) [13]. Complete responses were also more frequently observed in the pembrolizumab group than in the placebo group (11.3% vs. 3.1%). Moreover, the pembrolizumab group showed deeper responses than in the placebo group (median change from baseline, 65% versus 49%; ≥80% decrease from baseline, 32.3% vs. 14.8%). Grade 3 or worse adverse events occurred in 57.1% of the pembrolizumab group versus 57.4% in the placebo group. These interim results of KEYNOTE-811 led to FDA accelerated approval to adding pembrolizumab to trastuzumab and chemotherapy in the first-line treatment for patients with HER2 positive AGC. Results of OS and PFS (dual primary endpoints) are awaited.

Furthermore, the phase II trial of margetuximab, an Fc-optimized, anti-HER2 monoclonal antibody with increased affinity, demonstrated favorable results when combined with pembrolizumab with ORR of 24% and DCR of 62% in AGC patients with HER2 IHC3+ tumor in a second-line setting [65]. The phase II/III MAHOGANY trial, evaluating the efficacy of margetuximab with INCMGA00012 (anti-PD-1 antibody) or MGD013 (anti-PD-1/anti-LAG3 antibody) plus chemotherapy in previously untreated AGC patients is underway (NCT04082364). Most recently, in MAHOGANY trial Cohort A, margetuximab with INCMGA00012 showed ORR of 52.4% [66], warranting further evaluations in the ongoing trial.

In a phase I trial with HER2-positive solid tumors, ZW25 (an anti-HER2 bispecific antibody) was well tolerated and demonstrated ORR >30% in AGC [67]. A phase III trial (HERIZON-GEA-01) of ZW25 with chemotherapy with or without tislelizumab (anti-PD-1 antibody) as a first-line treatment for patients with HER2-positive AGC will be investigated.

Trastuzumab deruxtecan (T-DXd), an antibody-drug conjugate consisting of an anti-HER2 antibody, a topoisomerase I inhibitor, and a cleavable linker, was shown to improve response rate and OS in patients with HER2-positive AGC previously treated with trastuzumab-containing chemotherapy compared with third-line or later-line [68]. T-DXd activated dendritic cells, increased the expression of MHC class I in tumor cells, and enhanced the antitumor response to PD-1 blockade in the murine model [69]. An ongoing phase Ib/II trial, DESTINY-Gastric03, investigates several combinations of T-DXd with checkpoint inhibitors or other cytotoxic chemotherapies (NCT04379596).

### 3.4. Anti-PD-1 Antibody Plus Anti-CTLA4 Antibody

Cytotoxic T lymphocyte antigen 4 (CTLA-4), a key negative regulator of T-cells other than PD-1/PD-L1, restricts the antitumor immune response. Anti-CTLA-4 antibody binds to CTLA-4 on activated T cells and prevents T cell inactivation in lymph nodes during the initial stage of cancer-immunity cycles, while anti-PD-1/PD-L1 antibodies inhibit T cell inactivation in tumor tissue during the effector phase. Anti-CTLA-4 antibody also binds to CTLA-4 on regulatory T cells (one of the immune suppressive cells) and eliminates them by ADCC in tumor tissue [70,71,72]. The anti-PD-1/PD-L1 antibody plus anti-CTLA-4 antibody combination is expected to have a synergistic effect due to these different mode of actions between the two antibodies toward the tumor immune microenvironment. Indeed, the combination therapy with ipilimumab (anti-CTLA-4 antibody) and nivolumab has demonstrated antitumor activities in several malignancies [73,74,75,76,77]. In the AGC cohort of CheckMate-032, ORR was higher in patients with 1 mg/kg nivolumab plus 3 mg/kg ipilimumab (24%) than in those with 3 mg/kg nivolumab (12%) or 3 mg/kg nivolumab plus 1 mg/kg ipilimumab (8%) [78]. However, in the subsequent phase III CheckMate-649 trial, the enrollment to the cohort of 1 mg/kg nivolumab plus 3 mg/kg ipilimumab was closed earlier than chemotherapy plus nivolumab or chemotherapy alone arms. First results of nivolumab plus ipilimumab versus chemotherapy arms have been just recently reported [79]. Nivolumab plus ipilimumab did not improve OS compared with chemotherapy in patients with PD-L1 CPS ≥ 5 (median 11.2 months vs. 11.6 months; HR ≥ 0.89, *p* ≥ 0.2302); OS in all randomized patients was not statistically tested. Moreover, PFS benefit was not observed with nivolumab plus ipilimumab versus chemotherapy in either CPS ≥ 5 (median 2.8 months vs. 6.3 months) or any of the randomized populations (median 2.8 months vs. 6.1 months). ORR was lower with nivolumab plus ipilimumab versus chemotherapy (27% vs. 47% in CPS ≥ 5, 23% vs. 47% in all randomized populations), although duration of response was longer in both CPS ≥ 5 (13.2 months vs. 6.9 months) and all randomized populations (13.8 months vs. 6.8 months). Meanwhile, longer OS (not reached vs. 10.0 months, HR ≥ 0.28) and higher ORR (70% vs. 57%) with nivolumab plus ipilimumab compared with chemotherapy were observed in all randomized patients with MSI-H tumors. Grade 3 or 4 TRAEs were reported in 38% of patients with nivolumab plus ipilimumab including increased lipase (7%), increased amylase (4%), and increased ALT/AST (4% each) versus 46% of patients with chemotherapy. Serious TRAEs (23% vs. 12%) and TRAEs leading to discontinuation (17% vs. 10%) were frequently observed with nivolumab plus ipilimumab versus chemotherapy. Currently, a phase III trial (ATTRACTION-6), investigating nivolumab plus 1 mg/kg ipilimumab plus chemotherapy compared with chemotherapy, is ongoing in Asian countries.

### 3.5. Anti-PD-1 Antibody Plus Multikinase Inhibitors

Previous preclinical studies reported that inhibition of the VEGF pathway controlled tumor growth and inhibited the infiltration of immune suppressive cells, such as regulatory T cells, tumor-associated macrophages, and myeloid-derived suppressor cells, while it increased the mature dendritic cell fraction [80]. Multikinase inhibitors of VEGF receptors and other receptor tyrosine kinases, such as regorafenib or lenvatinib, substantially decreased immune suppressive cells and enhanced antitumor activity of PD-1 inhibitors in the in vivo models [81,82,83]. Furthermore, immune suppressive cells have been reported to be associated with rapid progression during ICIs, called hyperprogressive disease (HPD) [84]. Thus, targeting immune suppressive cells with multikinase inhibitors is expected to reduce HPD as well as enhance antitumor activity of ICIs. A phase Ib trial of regorafenib plus nivolumab demonstrated that ORR was 44% and median PFS was 5.6 months for AGC patients [85]. Notably, three of seven AGC patients refractory to previous anti-PD-1 antibodies achieved an objective response with this combination, supporting the concept of overcoming resistance of PD-1 blockade with regorafenib. A phase III trial (INTEGRATEIIb) of regorafenib and nivolumab combination compared with standard chemotherapy in third- or later-line treatment for AGC is being investigated (NCT04879368). In this trial, patients with prior ICIs included in stratification factors can be enrolled. In addition, a Japanese phase II trial of lenvatinib plus pembrolizumab demonstrated promising results in terms of antitumor efficacy, with ORR of 69% for AGC patients at first- or second-line [86]; however, another phase II trial of lenvatinib plus pembrolizumab (LEAP-005) found only 10% ORR in the AGC cohort [87]. The efficacy results in these trials are preliminary in nature as they were non-randomized phase II trials. Currently, a further phase III (LEAP-015) of lenvatinib plus pembrolizumab plus chemotherapy, followed by lenvatinib plus pembrolizumab versus chemotherapy for AGC in the first-line setting, is ongoing (NCT04662710). In the first-line setting, another phase III trial of camrelizumab (anti-PD-1 antibody) plus chemotherapy sequenced by apatinib with or without camrelizumab versus chemotherapy for AGC is also underway (NCT03813784). In the perioperative setting, we are currently conducting a phase II trial of lenvatinib with pembrolizumab in the neoadjuvant/adjuvant treatment for gastric cancer (NCT04745988).

### 3.6. CAR-T

Chimeric antigen receptor T (CAR-T) cell therapies have been enthusiastically investigated in solid tumors as well as hematological malignancy. A preclinical study indicated that in CLDN18.2-positive GC patient-derived tumor xenograft models, CLDN18.2-specific CAR-T cells achieved partial or complete tumor elimination [88]. Most recently, a phase I study of CLDN18.2-targeted CAR-T cell therapy demonstrated promising results with ORR of 48.6% with manageable safety profiles in gastrointestinal cancer patients [89]. AGC patients in third- or later-line settings at the dose of 2.5 × 10^8^ CAR-T cells achieved ORR of 61.1% with a median for PFS of 5.6 months and 9.5 months for OS. Additional studies are currently ongoing (NCT04400383 and NCT04467853).

## 4. Conclusions

In the first-line setting, recent pivotal clinical trials of anti-PD-1 antibodies plus conventional agents demonstrated clinical activity for both HER2-negative and HER2-positive AGC. These results have changed the standard of care in the first-line setting for AGC. However, given the greater efficacy of nivolumab plus chemotherapy in HER2-negative AGC with higher PD-L1 expression (CPS ≥ 5), it remains unclear whether this combination could be adopted irrespective of PD-L1 CPS or only for CPS ≥ 5 population. As described above, FDA and regulatory agencies in Asian countries (Japan, Korea, and China) have approved the combination of nivolumab and chemotherapy irrespective of PD-L1 CPS, while EMA and NCCN guidelines have approved or recommended it for AGC with CPS ≥ 5. We believe that PD-L1 CPS and MSI status should be investigated as much as possible, as these biomarkers were associated with clinical outcomes. Furthermore, considering that TRAEs increased in anti-PD-1 antibodies plus chemotherapy compared with conventional agents, chemotherapy alone should be an option with consideration for a patient’s general condition, complications such as autoimmune disease, and family support, especially in patients with CPS < 5.

Considering that a limited number of patients achieved clinical benefit of ICIs, the development of new immunotherapy is urgently needed. Currently, ICIs plus other targeted agents such as multikinase inhibitors and CLDN18.2-specific CAR-T cell therapies seem to be promising in early clinical trials, warranting further evaluations in subsequent studies.

## Figures and Tables

**Table 1:** This table shows pivotal clinical trials of anti-PD-1/PD-L1 therapies for gastric cancer.

Line	Phase	Trial	Region	Patient Selection (Cohort)	Arms (Regimen)	Primary Endpoint	OS			PFS			ORR	
							Med	HR	*p*	Med	HR	*p*	%	*p*
monotherapy														
3rd or later	III	ATTRACTION-2	Asia	all	Nivo	OS	5.26	0.63	<0.0001	1.61	0.60	<0.0001	11.2	-
					Placebo		4.14			1.45			0	-
3rd or later	II	KEYNOTE-059	Global	all	Pemb	ORR (all & CPS ≥ 1)	5.6	-	-	2.0	-	-	11.6	-
				CPS ≥ 1	Pemb		5.8	-	-	2.1	-	-	15.5	-
3rd	III	JAVELIN 300	Global	all	Avel	OS	4.6	1.1	0.81	1.4	1.73	>0.99	2.2	-
					physician’s choice		5.0			2.7			4.3	-
2nd	III	KEYNOTE-061	Global	CPS ≥ 1	Pemb	OS & PFS	9.1	0.82	0.0421	1.5	1.27	-	16	-
					PTX (80 mg/m^2^)		8.3			4.1			14	-
1st	III	KEYNOTE-062	Global	CPS ≥ 1 (HER2-neg)	Pemb	OS (CPS ≥ 1) (CPS ≥ 10) PFS (CPS ≥ 1)	10.6	0.91	-	2	1.66	-	15	-
					XP/FP		11.1			6.4			37.2	
				CPS ≥ 10 (HER2-neg)	Pemb		17.4	0.69	-	2.9	1.1	-	25	-
					XP/FP		10.8			6.1			38	
1st	III	JAVELIN 100	Global	all (HER2-neg)	FOLFOX/XELOX → Avel	OS (all & TPS ≥ 1)	10.4	0.91	0.1779	3.2	1.04	-	13.3	-
					FOLFOX/XELOX → cont		10.9			4.4			14.4	
				TPS ≥ 1	FOLFOX/XELOX → Avel		16.2	1.13	0.6352	4.1	1.04	-	-	-
					FOLFOX/XELOX → cont		17.7			9.7			-	
2nd or later	II	KEYNOTE-158	Global	MSI-H/dMMR GC	Pemb	ORR	NR	-	-	11	-	-	45.8	-
				TMB-H	Pemb		11.7	-	-	2.1	-	-	29	-
				TMB-H (not MSI-H)			-	-	-	-	-	-	28	-
				Not-TMB-H			12.8	-	-	2.1	-	-	6	-
2nd or later	I	GARNET cohort F	Global	dMMR or POLEmut non-endometrial solid tumors	Dostarlimab	ORR	-	-	-	-	-	-	38.7	-
+chemotherapy														
1st	III	KEYNOTE-062	Global	CPS ≥ 1 (HER2-neg)	Pemb + XP/FP	OS (CPS ≥ 1) (CPS ≥ 10) PFS (CPS ≥ 1)	12.5	0.85	0.05	6.9	0.84	0.04	48.6	-
					XP/FP		11.1			6.4			37.2	
				CPS ≥ 10 (HER2-neg)	Pemb + XP/FP		12.3	0.85	0.16	5.7	0.73	-	53	-
					XP/FP		10.8			6.1			38	
1st	III	CheckMate-649	Global	CPS ≥ 5 (HER2-neg)	XELOX/FOLFOX + Nivo	OS & PFS (CPS ≥ 5)	14.4	0.71	<0.0001	7.7	0.68	<0.0001	60	-
					XELOX/FOLFOX		11.1			6			45	-
				CPS ≥ 1 (HER2-neg)	XELOX/FOLFOX + Nivo		14	0.77	<0.0001	7.5	0.74	-	60	-
					XELOX/FOLFOX		11.3			6.9			46	-
				All (HER2-neg)	XELOX/FOLFOX + Nivo		13.8	0.8	0.0002	7.7	0.77	-	58	-
					XELOX/FOLFOX		11.6			6.9			46	-
1st	III	ATTRACTION-4	Asia	all (HER2-neg)	XELOX/SOX + Nivo	OS & PFS	17.5	0.90	0.257	10.5	0.68	0.0007	57.5	0.0088
					XELOX/SOX		17.2			8.3			47.8	
1st	III	ORIENT 16	China	CPS ≥ 5 (HER2-neg)	XELOX + Sint	OS (CPS ≥ 5 & all)	18.4	0.660	0.0023	7.7	0.628	0.0002	-	-
					XELOX		12.9			5.8			-	-
				All (HER2-neg)	XELOX + Sint		15.2	0.766	0.0090	7.1	0.636	<0.0001	58.2	-
					XELOX		12.3			5.7			48.4	-
+HER2-taregeted therapy														
1st	III	KEYNOTE-811	Global	HER2-pos	FP/XELOX + Tmab + Pemb	OS & PFS	-	-	-	-	-	-	74.4	0.00006
					FP/XELOX + Tmab		-	-	-	-	-	-	51.9	
1st	II/III	MAHOGANY Cohort A	Global	HER2 3+, CPS ≥ 1, non-MSI-H	Margetuximab+ Retifanlimab	ORR	NR	-	-	6.4	-	-	52.5	-
+anti-CTLA4 antibody														
2nd or later	I/II	CheckMate-032	Global	esophagogastric cancer	Nivo 3 mg/kg	ORR	6.2	-	-	1.4	-	-	12	-
					Nivo 1 + Ipi 3		6.9	-	-	1.4	-	-	24	-
					Nivo 3 + Ipi 1		4.8	-	-	1.6	-	-	8	-
1st	III	CheckMate-649	Global	CPS ≥ 5	Nivo 1 + Ipi 3	OS (CPS ≥ 5)	11.2	0.89	0.2302	2.8	1.42	-	27	-
					XELOX/FOLFOX		11.6			6.3			47	-
				all	Nivo 1 + Ipi 3		11.7	0.91	-	2.8	1.66	-	23	-
					XELOX/FOLFOX		11.8			7.1			47	-
+multikinase inhibitor														
3rd or later	Ib	REGONIVO	Japan	GC	Rego + Nivo	DLT	12.3	-	-	5.6	-	-	44	-
1st or 2nd	II	EPOC1706	Japan	all	Lenva + Pemb	ORR	NR	-	-	7.1	-	-	69	-
3rd or later	II	LEAP-005	Global	GC	Lenva + Pemb	ORR	5.9	-	-	2.5	-	-	10	-
CAR-T														
2nd or later	I	CT041-CG400	China	all	CT041	Safety and tolerability	-	-	-	-	-	-	48.6	-
				GC*	CT041		9.5	-	-	5.6	-	-	57.1	-

* Dose level of 2.5 × 10^8^ for at least 2 prior lines of therapy. Abbreviations: OS: overall survival; PFS: progression-free survival; ORR: objective response rate; med: median (months); HR: hazard ratio; p: *p* value; CPS: PD-L1 combined positive score; TPS: tumor proportion score; GC: gastric cancer; Pemb: pembrolizumab; Nivo: nivolumab; Sint: sintilimab; Avel: avelumab; cont: continuation of the same chemotherapy; PTX: paclitaxel; Nivo1: nivolumab 1 mg/kg; Nivo3: nivolumab 3 mg/kg; Ipi1: ipilimumab 1 mg/kg; Ipi3: ipilimumab 3 mg/kg; Rego: regorafenib; Lenva: lenvatinib; DLT: dose limiting toxicity.
